# Functional status and quality of life in post‐COVID‐19 patients two to three weeks after hospitalization: A cross‐sectional study

**DOI:** 10.1002/hsr2.1510

**Published:** 2023-08-22

**Authors:** Merita Qorolli, Samire Beqaj, Dafinë Ibrahimi‐Kaçuri, Ardiana Murtezani, Valon Krasniqi, Amra Mačak Hadžiomerović

**Affiliations:** ^1^ Faculty of Medicine, Physiotherapy Branch University of Prishtina Prishtina Republic of Kosovo; ^2^ University Clinical Center of Kosovo Prishtina Republic of Kosovo; ^3^ Faculty of Medicine University Fehmi Agani Gjakova Republic of Kosovo; ^4^ Faculty of Health Studies University of Sarajevo Sarajevo Bosnia and Herzegovina

**Keywords:** COVID‐19, dyspnea, physical functional performance, quality of life

## Abstract

**Background and Aims:**

Extended hospitalization due to coronavirus disease 2019 (COVID‐19) is associated with residual musculoskeletal and functional deficits lasting even 6 months after discharge; therefore, it is crucial that post‐hospitalized patients are promptly assessed. The aim of this study was to identify post‐COVID‐19 patients' functional status and quality of life, as well as to investigate their inter‐relatedness 2–3 weeks after hospital discharge.

**Methods:**

The study included 39 post‐COVID‐19 patients previously hospitalized in the Clinic for Infectious Diseases at the University Clinical Center of Kosovo (UCCK) from August to December 2021. Physiotherapeutic assessment encompassed socio‐demographic and clinical data including Short Physical Performance Battery (SPPB) for physical functional performance, hand grip strength, 6‐min Walk Test (6MWT) for aerobic capacity and endurance, EuroQol 5‐Dimension 5‐Level (EQ‐5D‐5L) for quality of life, Visual Analogue Scale (VAS) for pain, Borg CR10 for dyspnea, peripheral oxygen saturation and heart rate. Descriptive statistics, Pearson correlation, and multiple linear regression analysis were utilized for data processing.

**Results:**

The median (interquartile range [IQR]) for Borg CR10, VAS pain scale, total SPPB, grip strength, and 6MWT were 1 (0–3), 3 (1–6), 9 (8–10), 30.5 (23.2–43.5) kg, 344.5 (312.7–381.7) m respectively, while the mean (SD) for EQ‐5D‐5L index value was 0.7 (0.2). The strongest and most significant correlation was depicted between SPPB total score and its subscales, followed by correlation with EQ‐5D‐5L (*r* = 0.719, *p* < 0.001), grip strength (*r* = 0.612 *p* < 0.001), Borg CR10 (*r* = −0.515, *p* = 0.001), 6MWT (*r* = 0.416, *p* = 0.02), and VAS scale (*r* = −0.343, *p* = 0.03). Using the multiple regression analysis, the grip strength, Borg‐CR10, and 6MWT were found to be strongly predictive of SPPB total score.

**Conclusion:**

In post‐COVID‐19 patients' functional status and quality of life were impaired 2–3 weeks following hospitalization. SPPB showed the most frequent and significant correlation with other variables, hence it should be considered as one of the primary screening tools.

## Keypoints

- Based on the heterogeneity of symptom presentation and clinical course, there is a need to carefully monitor for signs and symptoms in individuals with postcoronavirus disease 2019 (COVID‐19) conditions, especially in previously hospitalized patients.
- There are no established core outcome measures to be applied in the framework of physical therapy for post‐COVID‐19 conditions.
- In our study, post‐COVID‐19 patients had impaired functional status and quality of life, as early as two weeks following hospitalization.
- We recommend that the Short Physical Performance Battery (SPPB) be used in the future as one of the primary screening tools for post‐COVID‐19 patients following hospitalization.

## 1 INTRODUCTION

Currently, over 761 million coronavirus disease 2019 (COVID‐19) cases have been verified worldwide.^1^ The depicted COVID‐19 severity pattern has been shown as asymptomatic infection, symptomatic infection with isolation at home, symptomatic infection requiring hospitalization, or symptomatic infection necessitating critical care and ventilator support.^2^ Available scientific literature indicates that patients due to COVID‐19 besides clinical symptoms^3^ also show an impaired functional status,^4^ and quality of life^5^

The estimated requirement for hospitalization increases proportionally with age reaching 9.2% for infected individuals older than 60 years.^6^ It has been estimated that up to 50% of COVID‐19 hospital patients may need continued care to ameliorate their long‐lasting consequences.^7^ Prolonged hospitalization and immobility due to COVID‐19 are associated with residual musculoskeletal and functional deficits, including fatigue, muscle weakness, and polyneuropathy,^8^ which in severe forms of the disease requiring intensive care, last even 6 months after discharge.^9^ Bearing in mind that the symptoms which are the consequences of COVID‐19 decrease physical function and deteriorate quality of life, it is crucial that post‐hospitalized patients are promptly assessed.^10^

It is stipulated that post‐COVID‐19 conditions could initially be identified at least 4 weeks after the onset of infection.^11^ Although there exist a few studies describing the physical function of post‐COVID‐19 patients upon hospital discharge, as well as exercise tolerance and quality of life from 2 to 8 weeks following hospital discharge,^12^ the level of functional abilities of post‐COVID‐19 patients during the first month after hospital discharge has not been sufficiently investigated.

Considering the paucity in the literature regarding the functional status and quality of life of patients following COVID‐19 infection, the aim of this study was to identify post‐COVID‐19 patients' functional status and quality of life, as well as to investigate whether functional status is related to quality of life, 2–3 weeks after hospital discharge.

## 2 METHODS

### 2.1 Study design and subjects

**Figure 1:**
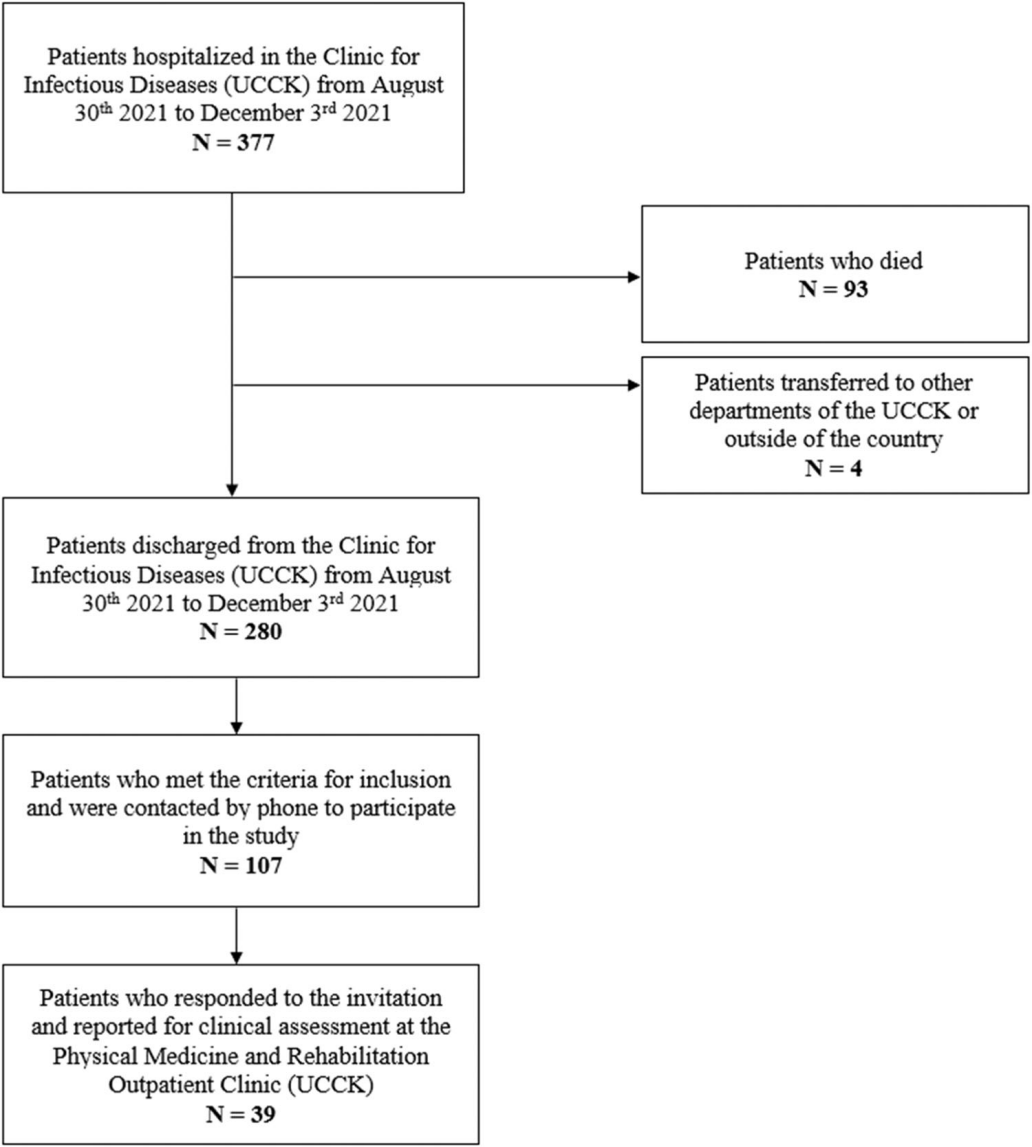
Chronology of patient inclusion.

This observational cross‐sectional study followed the Strengthening the Reporting of Observational studies in Epidemiology (STROBE) guidelines.^13^ The study included patients who survived COVID‐19 belonging to age 18 years or above, of both sexes, previously hospitalized in the Clinic for Infectious Diseases at the University Clinical Center of Kosovo (UCCK) from August 30, 2021 to December 3, 2021. A positive polymerase chain reaction (PCR) test for severe acute respiratory syndrome coronavirus 2 (SARS‐CoV‐2) was used for diagnosis of COVID‐19. Out of total of 377 hospitalized patients, 93 died, four were transferred to other departments of the UCCK or outside of the country, while 280 patients were discharged from the clinic. Based on the patient's history and discharge letter, as well as after consideration of exclusion criteria, that is, medical diagnoses the patient presented with at the time of discharge from hospital (moderate or severe heart disease (III or IV class, according to the functional classification by the New York Heart Association), chronic lung disease, chronic neurological diseases, renal insufficiency, cognitive deficit, acute diseases and injuries of the osteomuscular system, including the acute phase of rheumatological disorders and abnormality of the spinal disc, and inability to walk), 107 patients met the criteria for inclusion in the study. These patients were contacted by phone to report for clinical assessment at the Physical Medicine and Rehabilitation Outpatient Clinic at UCCK 2–3 weeks after hospital discharge. From the total number of contacted patients, 39 responded to the invitation for assessment. Figure 1 provides a diagram of the chronology of patient inclusion. A signed informed consent for involvement in the study was provided by all patients. For conduction of the study, permission from the Ethics Committee of the UCCK was obtained, protocol no. 1233, and the Declaration of Helsinki, as revised in 2013, was followed.

### 2.2 Materials and procedure

Patients underwent a physiotherapeutic assessment in the presence of a Physical Medicine and Rehabilitation physician. The physiotherapeutic assessment utilized in this study was designed based on Royal Dutch Society for Physical Therapy (KNGF) position statement for COVID‐19.^14^ Physiotherapeutic assessment included gathering of socio‐demographic data, determining the degree of pain, fatigue and dyspnea, peripheral oxygen saturation (SPO_2_) and heart rate (HR), functional status including physical function of lower extremities and balance, strength in upper extremity (hand grip strength), and aerobic capacity and endurance using the following instruments.

For assessment of pain during the week before evaluation a Visual Analogue Scale (VAS) was used.^15^ Borg CR10 scale of perceived exertion^16^ was used for the diagnosis of shortness of breath and dyspnea at rest, during, as well as following physical exertion. The scale ranges from 0 to 10, corresponding to different experiences of dyspnea and fatigue by the patient, 0 representing the absence of fatigue and 10 indicating an extremely severe, maximal fatigue. The Borg rating of perceived exertion was found to be a reliable measure of fatigue.^17^

Peripheral oxygen saturation (SpO_2_) and heart rate (HR) were measured at rest, during, and following the assessment of physical exertion with a portable pulse oximeter (Mod. No. OXY 200; Microlife AG).

#### 2.2.1 Physical function

The Short Physical Performance Battery (SPPB)^18^ was used for the assessment of balance, functional mobility, and lower extremity muscle strength during standing, sitting, and walking. SPPB consists of a balance test (the ability of a patient to stand with feet next to each other, in semi‐tandem and tandem position), a gait speed test (the time needed to walk a distance of 3 m), and a chair stand test (the time needed to repeatedly get up from the chair and sit down on the chair five times). The maximum possible score is 12, with each test score ranging from 0 to 4. Scores 0–3 indicate severe physical function disability, 4–6 low function, 7–9 intermediate function, while 10–12 normal function.^18^, ^19^ The SPPB is a valid and frequently used measure with a low SPPB score predicting falls, loss of independence, and mortality.^18^

A calibrated hydraulic dynamometer was used to assess the maximum hand grip strength, as an indicator of muscle strength and function. The recommendations of the American Society of Hand Therapists were followed, and the dynamometer was used in the “2” position.^20^ For interpretation of grip strength we used the normative reference values according to Wang et al.^21^

#### 2.2.2 Aerobic capacity and endurance

The 6‐min walk test (6MWT) was used to assess aerobic capacity and endurance. For application of the test guidelines developed by the American Thoracic Society^22^, ^23^ were followed. The distance walked during the 6‐min time is used as the outcome to assess performance. For the calculation of predicted reference walking distance, the gender‐ and anthropometric‐specific regression equations were used. For patients aged 40 or more, equations by Enright and Sherill^24^ were used, whereas for those younger than 40 years, equations by Gibbons et al.^25^ were used.

#### 2.2.3 Quality of life

Questionnaire EuroQOL‐5 Dimension‐5L (EQ‐5D‐5L)^26^ was utilized for the evaluation of quality of life (QoL). It is a generic instrument widely used for a variety of health conditions consisting of the descriptive system and VAS scale (EQ‐VAS). The descriptive system includes five domains: mobility, self‐care, usual activities, pain/discomfort, and anxiety/depression, with five levels of “problem” response options for each domain, ranging from no problem to extreme problem.^26^ It was required from the patient to indicate his/her health status by ticking the most appropriate answer in each of the five domains. The patient's health status as measured by this instrument is a combination of the given level for each domain, resulting in a five‐digit value corresponding to a total of 3125 health conditions. The index score ranges from −0.224 to 1, where negative values correspond to a health state worse than death, value 0 indicates a health state equivalent to death, while increasing positive values represent a better health state, with value 1 reflecting full health.^27^ The EQ‐VAS scale is a visual analogue scale consisting of values presented vertically ranging from 0, representing the worst imaginable health, up to 100, meaning the best imaginable health, thus corresponding to patients' subjective global comprehension of their health.

#### 2.2.4 Statistical analysis

The IBM‐SPSS Statistics 24 software was used to perform the statistical analysis. Demographic and clinical data were presented using descriptive statistics. For categorical variables, frequency analysis was used. The normality of distribution was assessed using the Shapiro–Wilk test. Normally distributed data were presented using the mean and standard deviation (SD), while not normally distributed data with median, as well as 25th and 75th interquartile range (IQR). The Pearson correlation coefficient was used to analyze the relation between the studied variables. The contribution of the tested variables on SPPB and EQ‐5D‐5L was explained using multiple linear regression analysis which covariates were selected based on theoretical considerations and correlation analyses. The confidence level was 95% (0.05), all the tests were two‐sided, and the significance level was set at *p* < 0.05 for all analyses.

## 3 RESULTS

### 3.1 Demographic data

In this study participated 39 post‐COVID‐19 subjects, 22 males (56.4%) and 17 females (43.6%). Mean age was 50.15 (12.20). Median duration of hospitalization as a result of COVID‐19 was 10 (7–14) days, whereas the median time frame between subjects' discharge from hospital and physiotherapeutic assessment was 16 (14–18) days. Table 1 shows collected demographic data.

**Table 1:** Demographic data (*N* = 39).

Variable	Mean (SD)	Median (IQR)
Age	50.1 (12.2)	
Duration of hospitalization (days)		10 (7–14)
Period between hospital discharge and assessment (days)		16 (14–18)

	*n*	%
Gender		
Male	22	56.4
Female	17	43.6
Level of education		
No education	2	5.1
Elementary	8	20.5
Secondary	19	48.7
University	10	25.6
Employment status		
Employed	17	43.6
Unemployed	22	56.4
Retired		
Marital status		
Married	31	79.5
Single	4	10.3
Divorced	1	2.6
Widowed	3	7.7
Tobacco consumption		
Yes	2	5.1
No	37	94.9

Abbreviations: IQR, interquartile range; SD, standard deviation.

### 3.2 Clinical data

Clinical data are presented in Table 2. Subjects' mean heart rate was 90.3 (14.0), SpO_2_ was 95.8 (1.8), while blood pressure 131.9 (16.2)/86.7 (11.4). Resting perceived exertion or dyspnea measured by Borg CR10 was 1 (0–3), corresponding to very weak dyspnea. Pain intensity assessed using VAS scale was 3 (1–6), which is analogous to light pain. The median for the total SPPB score, which encompasses the scores of all three subscales was 9 (8–10). The maximal possible score for each subscale is 4. The balance ability was found to have the highest score of 4 (4–4), followed by gait speed of 4 (3–4), while the chair stand test showed the lowest score of 1 (1–2). Grip strength was derived from mean value of right and left hands, and the median grip strength was 30.5 (23.2–43.5). In total 30 subjects underwent 6MWT. The remaining nine subjects declared unable or unwilling to do the test. The median walked distance was 344.5 (312.7–381.7). None of the subjects reached their reference 6MWT distance value with a median percentage of covered predicted distance being 54 (47.4–58.6). Subjects showed an impaired health quality of life following hospitalization due to COVID‐19. The mean EQ‐5D‐5L index value was 0.7 (0.2), while on EQ‐VAS scale subjects reported a mean score of 66.8 (16.3). The most common problems were reported with usual activities (74.3%), pain/discomfort (69.2%), and mobility (64.1%), followed by anxiety/depression (23.1%) and self‐care (20.5%). 7.7% of subjects were unable to perform usual activities at all, while 15.4% of them had major problems in performing the same activities. 10.3% of subjects had major problems with mobility, while 7.7% had severe pain/discomfort.

**Table 2:** Clinical data (*N* = 39).

Variable	Mean (SD)	Median (IQR)
HR	90.3 (14.0)	
SpO_2_	95.8 (1.8)	
Blood pressure		
Systolic	131.9 (16.2)	
Diastolic	86.7 (11.4)	
Borg CR10		1 (0–3)
VAS pain scale		3 (1–6)
SPPB		
Balance test		4 (4–4)
Gait speed test		4 (3–4)
Chair stand test		1 (1–2)
SPPB Total score		9 (8–10)
Grip strength (kg)		30.5 (23.2–43.5)
6MWT (m)		344.5 (312.7–381.7)
6‐MWT (m) % reference value		54.0 (47.4–58.6)
EQ‐5D‐5L	0.7 (0.2)	
EQ‐VAS	66.8 (16.3)	

Abbreviations: Borg CR10, scale of perceived exertion; EQ‐5D‐5L, EuroQol 5‐Dimension 5‐Level; HR, heart rate/minute; SpO_2_, blood oxygen saturation; SPPB, Short Physical Performance Battery; VAS pain scale, Visual Analogue Scale for assessment of pain; 6MWT, 6‐min walk test.

### 3.3 Correlation analysis

The results obtained by Pearson's correlation analysis are presented in Table 3. They demonstrated a significant correlation between VAS pain scale and grip strength (*r* = −0.459, *p* = 0.003), VAS scale and SPPB balance test (*r* = −0.326, *p* = 0.04), VAS scale and SPPB total score (*r* = −0.343, *p* = 0.03), and VAS scale and EQ‐5D‐5L (*r* = −0.465, *p* = 0.003). Borg CR10 was significantly associated with SPPB gait speed test (*r* = −0.602, *p* < 0.001), SPPB chair stand test (*r* = −0.405, *p* = 0.01), total SPPB score (*r* = −0.515, *p* = 0.001), and EQ‐5D‐5L (*r* = −0.350, *p* = 0.03). Grip strength demonstrated a significant correlation with SPPB balance test (*r* = 0.404, *p* = 0.01), SPPB gait speed test (*r* = 0.368, *p* = 0.02), SPPB chair to stand test (*r* = 0.745, *p* < 0.001), SPPB total score (*r* = 0.612, *p* < 0.001), as well as with EQ‐5D‐5L (*r* = 0.545, *p* < 0.001). A statistically significant relation was also found between SPPB total score and its subscales. SPPB total score was significantly correlated with EQ‐5D‐5L (*r* = 0.719, *p* < 0.001) and 6MWT (*r* = 0.416, *p* = 0.02). 6MWT also showed a significant correlation with the SPPB chair stand test (*r* = 0.367, *p* = 0.05).

**Table 3:** Correlation between assessed variables.

	Variable		1	2	3	4	5	6	7	8	9	10
1.	Duration of hospitalization (days)	*r*	1									
2.	VAS pain scale	*r*	**−0.327**	1								
		*p*‐Value	0.04									
3.	Borg CR10	*r*	−0.099	0.182	1							
		*p*‐Value	0.55	0.27								
4.	Grip strength	*r*	0.102	**−0.459**	−0.261	1						
		*p*‐Value	0.54	0.003	0.11							
5.	SPPB balance test	*r*	0.068	**−0.326**	−0.161	**0.404**	1					
		*p*‐Value	0.68	0.04	0.33	0.01						
6.	SPPB gait speed test	*r*	0.078	−0.294	**−0.602**	**0.368**	**0.585**	1				
		*p*‐Value	0.64	0.07	<0.001	0.02	<0.001					
7.	SPPB chair stand test	*r*	0.000	−0.233	**−0.405**	**0.745**	**0.385**	**0.513**	1			
		*p*‐Value	>0.99	0.15	0.01	<0.001	0.01	0.001				
8.	SPPB total score	*r*	0.060	**−0.343**	**−0.515**	**0.612**	**0.761**	**0.891**	**0.782**	1		
		*p*‐Value	0.72	0.03	0.001	<0.001	<0.001	<0.001	<0.001			
9.	6MWT	*r*	−0.117	0.013	−0.145	0.151	‐	0.315	**0.367**	**0.416**	1	
		*p*‐Value	0.54	0.94	0.44	0.42	‐	0.09	0.05	0.02		
10.	EQ‐5D‐5L	*r*	−0.037	**−0.465**	**−0.350**	**0.545**	**0.677**	**0.554**	**0.569**	**0.719**	0.103	1
		*p*‐Value	0.82	0.003	0.03	<0.001	<0.001	<0.001	<0.001	<0.001	0.6	

*Note*: Bold values are statistically significant at *p* values.

Abbreviations: Borg CR10, scale of perceived exertion; EQ‐5D‐5L, EuroQol 5‐Dimension 5‐Level; HR, heart rate; SPPB, Short Physical Performance Battery; SpO_2_, blood oxygen saturation; VAS pain scale, Visual Analogue Scale for assessment of pain; 6MWT, 6‐min walk test.

### 3.4 Regression analysis

The multiple regression model for the prediction of SPPB total score included variables VAS pain scale, Borg CR10, grip strength, 6MWT, and EQ‐5D‐5L. After statistically insignificant variables were removed, the model, including grip strength, Borg CR10, and 6MWT explained 64% of the variance in the total SPPB score (Table 4).

**Table 4:** Contribution of assessed (independent) variables on SPPB total score.

	Unstandardized coefficients		Standardized coefficients	*t*	Sig.	95.0% confidence interval for *B*	
	*B*	Std. error	*β*			Lower bound	Upper bound
(Constant)	6.447	0.809		7.966	<0.001	4.784	8.111
Grip strength	0.040	0.011	0.462	3.759	0.001	0.018	0.062
BORG CR10	−0.341	0.102	−0.411	−3.351	0.002	−0.550	−0.132
6MWT	0.005	0.002	0.287	2.394	0.02	0.001	0.009

*Note*: *R* ^2^ = 0.64, *p* < 0.001.

Abbreviations: Borg CR10, scale of perceived exertion; *R* ^2^, squared correlation coefficient; SPPB, Short Physical Performance Battery; 6MWT, 6‐min walk test.

Table 5 shows the regression model for the prediction of quality of life (EQ‐5D‐5L). The multiple regression included variables VAS pain scale, Borg CR10, grip strength, SPPB total score, and 6MWT. The SPPB total score resulted as the only statistically significant predictor and explained 52% of the variance in EQ‐5D‐5L.

**Table 5:** Contribution of SPPB total score on EQ‐5D‐5L.

	Unstandardized coefficients		Standardized coefficients	*t*	Sig.	95.0% confidence interval for *B*	
	*B*	Std. error	*β*			Lower bound	Upper bound
(Constant)	0.079	0.098		0.805	0.426	−0.119	0.277
SPPB total score	0.071	0.011	0.719	6.300	<0.001	0.048	0.094

*Note*: *R* ^2^ = 0.52, *p* < 0.001.

Abbreviations: EQ‐5D‐5L ‐ EuroQol 5‐Dimension 5‐Level; *R* ^2‐^Squared correlation coefficient; SPPB, Short Physical Performance Battery.

## 4 DISCUSSION

The literature has so far defined how the long‐lasting consequences of the COVID‐19 condition affect physical function, quality of life, and general health.^28^ It has been described that based on the heterogeneity of symptom presentation and clinical course, there is a need to carefully monitor for signs and symptoms in individuals with post‐COVID‐19 conditions,^29^ especially in previously hospitalized patients.^10^

Based on available studies, there are no established core outcome measures to be applied in the framework of physical therapy for post‐COVID‐19 condition,^30^ although there are several guidelines that stipulate the time frame of evaluation as well as which measures should be employed in post‐COVID‐19 patients.^10^, ^14^, ^30^, ^31^ As a matter of fact, most of the studies focusing on the investigation of physical performance, daily activities and quality of life in patients following COVID‐19, carried out assessments at later stages following hospital discharge.^32^ Therefore, the need for assessment at an earlier postdischarge stage has been emphasized. That being the case, the present study aimed to determine the functional status and quality of life in post‐COVID‐19 patients 2–3 weeks after hospitalization, as well as to investigate the association between measurements used for assessment of functional status and quality of life.

Regarding functional capacity, from the distance covered by the patients of our study during 6MWT, 346 (312.5–382.5) m, (54% of predicted value), it can be conveyed that aerobic STATUS and endurance were generally impaired, given that performance lower than 80% of predicted value is considered diminished.^24^ Other studies have also reported a decrease in aerobic performance in patients with long COVID‐19. In the study of Hazarika et al.^33^ and that of the COMEBAC Study Group,^34^ the assessment was performed at 3 and 4 months after discharge, with mean covered distance during 6MWT being 464 m and 480 m, respectively.^33^, ^34^ Similarly, other studies reported that a considerable number of post‐COVID‐19 patients showed impaired results in 6MWT as late as 5 and 6 months after hospital discharge.^9^, ^29^ Paneroni et al. did the evaluation at hospital discharge and presented that endurance was decreased, corresponding to 63% of the predicted normal value using the 1‐min sit‐to‐stand test,^35^ which has recently been shown that in patients following COVID‐19 can be used as an alternative in conditions where 6MWT cannot be performed.^36^ Nevertheless, in post‐COVID‐19 patients, the 6MWT has been shown as the most widely used test for the assessment of aerobic capacity.

We have found that overall physical performance was shown to be reduced with the median total SPPB score 9 (8–10). Moreover, 67% of patients had SPPB total score <10, while 18% of patients had SPPB total score <7, corresponding to severe physical disability and low function.^18^ The studies that have undertaken SPPB evaluation at hospital discharge have shown a higher level of disability, which is expected considering the sooner time of evaluation in comparison to our findings.^19^, ^37^ On the other hand, better performance was encountered in those assessed 3‐4 months after hospital discharge, with only 22.3% of patients scoring low on SPPB, indicating impaired physical functioning.^38^, ^39^

As an indicator of overall strength,^40^, ^41^ it is suggested that measuring the hand grip strength is important in COVID‐19 patients,^42^ as well as in later stages following COVID‐19 infection.^43^ Our study has shown that grip strength was diminished in 33% of patients, who performed lower than 80% of the reference value by Wang et al.^21^ Similar to our findings, decreased grip strength was found in both, the acute stage of infection^44^ and in the post‐COVID‐19 condition.^45^, ^46^

Our results showed that difficulties in endurance and physical performance were accompanied by dyspnea. Patients of our study reported a median Borg score of 1 (0–3), corresponding to very weak dyspnea.^16^ Our results agree with the findings of Halpin et al. who reported that 42.6% of post‐COVID‐19 patients, previously admitted to hospital wards, excluding intensive care unit (ICU), presented with breathing difficulty, the majority of whom had light dyspnea.^47^ On the other hand, dyspnea was described as moderate 1 month after hospital discharge,^48^ which is expected in patients with severe COVID‐19. The discrepancy of the latter^48^ with our study might be explained due to the fact that the inclusion criteria in our study excluded patients with severe comorbidities, thus none of our patients was admitted to ICU.

Areas of EQ‐5L‐5D that our patients reported most difficulties with were usual activities (74.3%), pain/discomfort (69.2%), and mobility (64.1%). Our results regarding pain/discomfort and mobility are in line with the findings of the systematic review and meta‐analysis of Malik et al.,^49^ who described these two domains were among the most frequently reported by post‐COVID‐19 patients 1–6 months after discharge. Pain/discomfort was also found to be most affected in the systematic review of Nandasena et al.^50^ on patients 15 days to 6 months following discharge from hospital due to COVID‐19. In our study, the least affected domain was self‐care (20.5%), which corresponds to existing literature.^49^, ^50^ Less profoundly were reported anxiety/depression (23.1%) and self‐care (20.5%). Knowing that health‐related quality of life has been shown to be reduced also in nonhospitalized patients even after 1 year, especially in areas of pain/discomfort and usual activities,^51^, ^52^ there is a need for long‐term follow‐up.

The correlation analysis showed that SPPB most frequently and significantly correlated with other tested variables. The strongest relation, as expected, was depicted between the total SPPB score and its subscales, followed by a significant correlation with EQ‐5D‐5L. There is a large paucity of literature regarding post‐COVID‐19 patients in terms of relations between different aspects of physical and functional performance, and quality of life. Nevertheless, a strong association between SPPB and EQ‐5D‐5L was reported in other populations with chronic diseases.^53^ Correlation between SPPB and grip strength has been widely explored and established in other populations,^54^, ^55^, ^56^ which has also been confirmed in our study. Similarly, Piquet et al. have found a correlation between grip strength and functional ability measured with Barthel Index,^57^ the measure which has been described as one of the most frequently used for evaluation of functional abilities, along with SPPB, 6MWT, and 1 min sit‐to‐stand.^4^ Notably, we have found SPPB to be significantly correlated with 6MWT, which has also been investigated in other non‐COVID‐19 studies.^58^, ^59^

Using the multiple linear regression analysis, the grip strength, Borg‐CR10, and 6MWT were found to be strongly predictive of SPPB total score, accounting for 64.0% of the total variance. The SPPB has been identified in the regression analysis as the only measure to reflect quality of life. Considering the practicability of SPPB, our results support the previous statements describing it as one of the simple and affordable tools.^14^, ^31^, ^60^

Our research presents one of the very few scientific studies on post‐COVID‐19 patients describing the level of functional status and quality of life 2–3 weeks following hospital discharge. However, our study has a few limitations. First, the rigorous exclusion criteria in our study contributed to the absence of patients with severe and critical courses of COVID‐19, thus resulting in a relatively homogenous sample size. Considering that none of our patients required the use of a ventilator, nor was admitted to ICU, our findings can be useful for posthospitalized COVID‐19 patients with mild to moderate courses of disease, while an application to other clinical groups or populations should be approached with caution. Second, there was a lack of pre‐hospitalization clinical data of our patients, thus limiting the recognition of the extent of COVID‐19's effect on their functional status and quality of life.

## 5 CONCLUSION

Considering that in our study, post‐COVID‐19 patients had impaired functional status and quality of life, we recommend that all posthospitalized COVID‐19 patients are assessed for physical function, aerobic capacity, endurance and quality of life as early as 2 weeks following hospitalization. Following this prompt assessment, the results allow for early identification of functional impairment and direct the implementation of a patient‐tailored physical therapy program, thus reducing the impact of long‐term sequelae experienced by people with COVID‐19.

Based on findings that SPPB most frequently and significantly correlated with other tested variables, as well as that SPPB was predicted by grip strength, Borg‐CR10, and 6MWT, we consider that the SPPB instrument best reflects the functional status of posthospitalized COVID‐19 patients. We recommend that SPPB is used in future as one of the primary screening tools in post‐COVID‐19 patients following hospitalization.

## AUTHOR CONTRIBUTIONS

**Merita Qorolli**: Conceptualization; formal analysis; funding acquisition; investigation; methodology; project administration; resources; validation; visualization; writing—original draft; writing—review & editing. **Samire Beqaj**: Conceptualization; data curation; formal analysis; investigation; methodology; resources; validation; visualization; writing—original draft; writing—review & editing. **Dafinë Ibrahimi‐Kaçuri**: Conceptualization; data curation; investigation; writing—review & editing. **Ardiana Murtezani**: Data curation; investigation; resources. **Valon Krasniqi**: Investigation; resources; writing—review & editing. **Amra Mačak Hadžiomerović**: Conceptualization; supervision.

## CONFLICTS OF INTEREST STATEMENT

The authors declare no conflicts of interest.

## TRANSPARENCY STATEMENT

The lead author Samire Beqaj affirms that this manuscript is an honest, accurate, and transparent account of the study being reported; that no important aspects of the study have been omitted; and that any discrepancies from the study as planned (and, if relevant, registered) have been explained.

## Data Availability

The data that support the findings of this study are available from the corresponding author upon reasonable request.

## References

[1] World Health Organization . Weekly epidemiological update on COVID‐19. Updated March 30, 2023. Accessed April 1, 2023. https://www.who.int/publications/m/item/weekly-epidemiological-update-on-covid-19---30-march-2023

[2] Barker‐Davies RM , O'Sullivan O , Senaratne KPP , et al. The Stanford Hall consensus statement for post‐COVID‐19 rehabilitation. Br J Sports Med. 2020;54(16):949‐959. 10.1136/bjsports-2020-102596 32475821 PMC7418628

[3] Wang Y , Wang Y , Chen Y , Qin Q . Unique epidemiological and clinical features of the emerging 2019 novel coronavirus pneumonia (COVID‐19) implicate special control measures. J Med Virol. 2020;92(6):568‐576. 10.1002/jmv.25748 32134116 PMC7228347

[4] Simonelli C , Paneroni M , Vitacca M , Ambrosino N . Measures of physical performance in COVID‐19 patients: a mapping review. Pulmonology. 2021;27(6):518‐528. 10.1016/j.pulmoe.2021.06.005 34284976 PMC8221906

[5] Ma YF , Li W , Deng HB , et al. Prevalence of depression and its association with quality of life in clinically stable patients with COVID‐19. J Affect Disord. 2020;275:145‐148. 10.1016/j.jad.2020.06.033 32658818 PMC7329672

[6] Menachemi N , Dixon BE , Wools‐Kaloustian KK , Yiannoutsos CT , Halverson PK . How many SARS‐CoV‐2‐Infected people require hospitalization? Using random sample testing to better inform preparedness efforts. J Public Health Manag Pract. 2021;27(3):246‐250. 10.1097/PHH.0000000000001331 33729203

[7] Yang T , Yan MZ , Li X , Lau EHY . Sequelae of COVID‐19 among previously hospitalized patients up to 1 year after discharge: a systematic review and meta‐analysis. Infection. 2022;50(5):1067‐1109. 10.1007/s15010-022-01862-3 35750943 PMC9244338

[8] Frota AX , Vieira MC , Soares CCS , et al. Functional capacity and rehabilitation strategies in Covid‐19 patients: current knowledge and challenges. Rev Soc Bras Med Trop. 2021;54:0. 10.1590/0037-8682-0789-2020 PMC7849325 33533821

[9] Sirayder U , Inal‐Ince D , Kepenek‐Varol B , Acik C . Long‐term characteristics of severe COVID‐19: respiratory function, functional capacity, and quality of life. Int J Environ Res Public Health. 2022;19(10):6304. 10.3390/ijerph19106304 35627841 PMC9141122

[10] Spruit MA , Holland AE , Singh SJ , Tonia T , Wilson KC , Troosters T . COVID‐19: interim guidance on rehabilitation in the hospital and post‐hospital phase from a European Respiratory Society‐ and American Thoracic Society‐coordinated international task force. Eur Respir J. 2020;56(6):2002197. 10.1183/13993003.02197-2020 32817258 PMC7427118

[11] Centers for Disease Control and Prevention . Post‐COVID conditions: Information for healthcare providers. Updated December 16, 2022. Accessed March 30, 2023. https://www.cdc.gov/coronavirus/2019-ncov/hcp/clinical-care/post-covid-conditions.html

[12] Araújo BTS , Barros AEVR , Nunes DTX , et al. Effects of continuous aerobic training associated with resistance training on maximal and submaximal exercise tolerance, fatigue, and quality of life of patients post‐COVID‐19. Physiother Res Int. 2023;28(1):e1972. 10.1002/pri.1972 36088642 PMC9539049

[13] von Elm E , Altman DG , Egger M , Pocock SJ , Gøtzsche PC , Vandenbroucke JP . The strengthening the reporting of observational studies in epidemiology (STROBE) statement: guidelines for reporting observational studies. JCE. 2008;61(4):344‐349. 10.1016/j.jclinepi.2007.11.008 18313558

[14] Royal Dutch Society for Physical Therapy (KNGF) , KNGF position statement: Physiotherapy recommendations in patients with COVID‐19, Amersfoort, The Netherlands, May, 2020.

[15] Hawker GA , Mian S , Kendzerska T , French M . Measures of adult pain: Visual Analog Scale for Pain (VAS Pain), Numeric Rating Scalenumeric rating scale for pain (NRS Pain), McGill pain questionnaire (MPQ), Short‐Form McGill Pain Questionnaire (SF‐MPQ), Chronic Pain Grade Scale (CPGS), Shortshort Form‐36 Bodily Pain Scale (SF‐36 BPS), and Measure of Intermittent and Constant Osteoarthritis Pain (ICOAP). Arthritis Care Res. 2011;63(suppl_11):S240‐S252. 10.1002/acr.20543 22588748

[16] Borg GAV . Psychophysical bases of perceived exertion. Med Sci Sports Ex. 1982;14(5):377‐381. 10.1249/00005768-198205000-00012 7154893

[17] Skinner JS , Hutsler E , Bergsteinova V , Buskirk ER . The validity and reliability of a rating scale of perceived exertion. Med Sci Sports Ex. 1973;5(2):94‐96. 4721013

[18] Guralnik JM , Simonsick EM , Ferrucci L , et al. A short physical performance battery assessing lower extremity function: association with self‐reported disability and prediction of mortality and nursing home admission. J Gerontol. 1994;49(2):M85‐M94. 10.1093/geronj/49.2.M85 8126356

[19] Paneroni M , Simonelli C , Saleri M , et al. Muscle strength and physical performance in patients without previous disabilities recovering from COVID‐19 pneumonia. Am J Phys Med Rehabil. 2021;100(2):105‐109. 10.1097/PHM.0000000000001641 33181531

[20] Mathiowetz V , Rennells C , Donahoe L . Effect of elbow position on grip and key pinch strength. J Hand Surg. 1985;10(5):694‐697. 10.1016/S0363-5023(85)80210-0 4045150

[21] Wang YC , Bohannon RW , Li X , Sindhu B , Kapellusch J . Hand‐Grip strength: normative reference values and equations for individuals 18 to 85 years of age residing in the United States. J Orthop Sports Phys Ther. 2018;48(9):685‐693. 10.2519/jospt.2018.7851 29792107

[22] ATS Committee on Proficiency Standards for Clinical Pulmonary Function Laboratories . ATS statement: guidelines for the six‐minute walk test. Am J Respir Crit Care Med. 2002;166(1):111‐117. 10.1164/ajrccm.166.1.at1102 12091180

[23] Erratum: ATS statement: guidelines for the six‐minute walk test. Am J Respir Crit Care Med. 2016;193(10):1185. 10.1164/rccm.19310erratum 27174486

[24] Enright PL , Sherrill DL . Reference equations for the six‐minute walk in healthy adults. Am J Respir Crit Care Med. 1998;158(5):1384‐1387. 10.1164/ajrccm.158.5.9710086 9817683

[25] Gibbons WJ , Fruchter N , Sloan S , Levy RD . Reference values for a multiple repetition 6‐minute walk test in healthy adults older than 20 years. J Cardiopulm Rehabil. 2001;21(2):87‐93. 10.1097/00008483-200103000-00005 11314289

[26] Herdman M , Gudex C , Lloyd A , et al. Development and preliminary testing of the new five‐level version of EQ‐5D (EQ‐5D‐5L). Qual Life Res. 2011;20(10):1727‐1736. 10.1007/s11136-011-9903-x 21479777 PMC3220807

[27] Devlin NJ , Shah KK , Feng Y , Mulhern B , van Hout B . Valuing health‐related quality of life: an EQ‐5D‐5L value set for England. Health Econ. 2018;27(1):7‐22. 10.1002/hec.3564 28833869 PMC6680214

[28] Montes‐Ibarra M , Oliveira CLP , Orsso CE , Landi F , Marzetti E , Prado CM . The impact of long COVID‐19 on muscle health. Clin Geriatr Med. 2022;38(3):545‐557. 10.1016/j.cger.2022.03.004 35868672 PMC8934728

[29] Wahlgren C , Divanoglou A , Larsson M , et al. Rehabilitation needs following COVID‐19: five‐month post‐discharge clinical follow‐up of individuals with concerning self‐reported symptoms. EClinicalMedicine. 2022;43:101219. 10.1016/j.eclinm.2021.101219 34901798 PMC8645256

[30] World Health Organization . Clinical management of COVID‐19: living guideline, (WHO/2019‐nCoV/clinical/2023.1). Licence: CC BY‐NC‐SA 3.0 IGO. January 13, 2023.

[31] Wells CL , Kegelmeyer D , Mayer KP , et al. APTA cross sections and academies recommendations for COVID‐19 core outcome measures. J Acute Care Phys Ther. 2022;13(2):62‐76. 10.1097/JAT.0000000000000172 35340890 PMC8939471

[32] de Oliveira Almeida K , Nogueira Alves IG , de Queiroz RS , et al. A systematic review on physical function, activities of daily living and health‐related quality of life in COVID‐19 survivors. Chronic Illn. 2023;19(2):279‐303. 10.1177/17423953221089309 35404175 PMC9006095

[33] Hazarika A , Mahajan V , Kajal K , et al. Pulmonary function, mental and physical health in recovered COVID‐19 patients requiring invasive versus non‐invasive oxygen therapy: a prospective follow‐up study post‐ICU discharge. Cureus. 2021;13(9):e17756. 10.7759/cureus.17756 34659969 PMC8493858

[34] Morin L , Savale L , Pham T , et al. Four‐Month clinical status of a cohort of patients after hospitalization for COVID‐19. JAMA. 2021;325(15):1525. 10.1001/jama.2021.3331 33729425 PMC7970386

[35] Paneroni M , Vogiatzis I , Bertacchini L , Simonelli C , Vitacca M . Predictors of low physical function in patients with COVID‐19 with acute respiratory failure admitted to a subacute unit. Arch Phys Med Rehabil. 2021;102(6):1228‐1231. 10.1016/j.apmr.2020.12.021 33529611 PMC7846883

[36] Peroy‐Badal R , Sevillano‐Castaño A , Torres‐Castro R , et al. Comparison of different field tests to assess the physical capacity of post‐COVID‐19 patients. Pulmonology. 2022;9(55):1‐8. 10.1016/j.pulmoe.2022.07.011 PMC9339971 36117103

[37] Belli S , Balbi B , Prince I , et al. Low physical functioning and impaired performance of activities of daily life in COVID‐19 patients who survived hospitalisation. Eur Respir J. 2020;56(4):2002096. 10.1183/13993003.02096-2020 32764112 PMC7411272

[38] Bellan M , Soddu D , Balbo PE , et al. Respiratory and psychophysical sequelae among patients with COVID‐19 four months after hospital discharge. JAMA Netwk Open. 2021;4(1):e2036142. 10.1001/jamanetworkopen.2020.36142 PMC7841464 33502487

[39] Baricich A , Borg MB , Cuneo D , et al. Midterm functional sequelae and implications in rehabilitation after COVID‐19: a cross‐sectional study. Eur J Phys Rehabil Med. 2021;57(2):199‐207. 10.23736/S1973-9087.21.06699-5 33565741

[40] Patrizio E , Calvani R , Marzetti E , Cesari M . Physical functional assessment in older adults. J Frailty Aging. 2020;10(2):1‐9. 10.14283/jfa.2020.61 33575703

[41] Beaudart C , Rolland Y , Cruz‐Jentoft AJ , et al. Assessment of muscle function and physical performance in daily clinical practice: a position paper endorsed by the European Society for Clinical and Economic Aspects of Osteoporosis, Osteoarthritis and Musculoskeletal Diseases (ESCEO). Calcif Tissue Int. 2019;105(1):1‐14. 10.1007/s00223-019-00545-w 30972475

[42] Cheval B , Sieber S , Maltagliati S , et al. Muscle strength is associated with COVID‐19 hospitalization in adults 50 years of age or older. J Cachexia Sarcopenia Muscle. 2021;12(5):1136‐1143. 10.1002/jcsm.12738 34363345 PMC8426913

[43] Del Brutto OH , Mera RM , Pérez P , Recalde BY , Costa AF , Sedler MJ . Hand grip strength before and after SARS‐CoV‐2 infection in community‐dwelling older adults. J Am Geriatr Soc. 2021;69(10):2722‐2731. 10.1111/jgs.17335 34124775 PMC8447376

[44] Kara Ö , Kara M , Akın ME , Özçakar L . Grip strength as a predictor of disease severity in hospitalized COVID‐19 patients. Heart & Lung: J Critical Care. 2021;50(6):743‐747. 10.1016/j.hrtlng.2021.06.005 PMC8192888 34217985

[45] Tanriverdi A , Savci S , Kahraman BO , Ozpelit E . Extrapulmonary features of post‐COVID‐19 patients: muscle function, physical activity, mood, and sleep quality. Irish J Med Sci. 2022;191(3):969‐975. 10.1007/s11845-021-02667-3 34080125 PMC8172250

[46] van Gassel RJJ , Bels J , Remij L , et al. Functional outcomes and their association with physical performance in mechanically ventilated coronavirus disease 2019 survivors at 3 months following hospital discharge: a cohort study. Crit Care Med. 2021;49(10):1726‐1738. 10.1097/CCM.0000000000005089 33967204 PMC8439632

[47] Halpin SJ , McIvor C , Whyatt G , et al. Postdischarge symptoms and rehabilitation needs in survivors of COVID‐19 infection: A cross‐sectional evaluation. J Med Virol. 2021;93(2):1013‐1022. 10.1002/jmv.26368 32729939

[48] Weerahandi H , Hochman KA , Simon E , et al. Post‐discharge health status and symptoms in patients with severe COVID‐19. J Gen Intern Med. 2021;36(3):738‐745. 10.1007/s11606-020-06338-4 33443703 PMC7808113

[49] Malik P , Patel K , Pinto C , et al. Post‐acute COVID‐19 syndrome (PCS) and health‐related quality of life (HRQoL)—a systematic review and meta‐analysis. J Med Virol. 2022;94(1):253‐262. 10.1002/jmv.27309 34463956 PMC8662132

[50] Nandasena HMRKG , Pathirathna ML , Atapattu AMMP , Prasanga PTS . Quality of life of COVID 19 patients after discharge: systematic review. PLoS One. 2022;17(2):e0263941. 10.1371/journal.pone.0263941 35171956 PMC8849513

[51] Tarazona V , Kirouchena D , Clerc P , Pinsard‐Laventure F , Bourrion B . Quality of life in COVID‐19 outpatients: a long‐term follow‐up study. J Clin Med. 2022;11(21):6478. 10.3390/jcm11216478 36362706 PMC9657247

[52] Parker M , Sawant HB , Flannery T , et al. Effect of using a structured pacing protocol on post‐exertional symptom exacerbation and health STATUS in a longitudinal cohort with the post‐COVID‐19 syndrome. J Med Virol. 2023;95(1):e28373. 10.1002/jmv.28373 36461167 PMC9878088

[53] Oh B , Cho B , Choi HC , et al. The influence of lower‐extremity function in elderly individuals' quality of life (QOL): an analysis of the correlation between SPPB and EQ‐5D. Arch Gerontol Geriat. 2014;58(2):278‐282. 10.1016/j.archger.2013.10.008 24275121

[54] Kudelka J , Geritz J , Welzel J , et al. What contributes most to the SPPB and its subscores in hospitalized geriatric patients: an ICF model‐based approach. BMC Geriatr. 2022;22(1):668. 10.1186/s12877-022-03358-z 35963992 PMC9375907

[55] Legrand D , Vaes B , Matheï C , Adriaensen W , Van Pottelbergh G , Degryse JM . Muscle strength and physical performance as predictors of mortality, hospitalization, and disability in the oldest old. J Am Geriatr Soc. 2014;62(6):1030‐1038. 10.1111/jgs.12840 24802886

[56] Muollo V , Tatangelo T , Ghiotto L , et al. Is handgrip strength a marker of muscle and physical function of the lower limbs? Sex differences in older adults with obesity. Nutr Metab Cardiovasc Dis. 2022;32(9):2168‐2176. 10.1016/j.numecd.2022.06.018 35850750

[57] Piquet V , Luczak C , Seiler F , et al. Do patients with COVID‐19 benefit from rehabilitation? Functional outcomes of the first 100 patients in a COVID‐19 rehabilitation unit. Arch Phys Med Rehabil. 2021;102(6):1067‐1074. 10.1016/j.apmr.2021.01.069 33548208 PMC7857995

[58] Oliveira JM , Spositon T , Cerci Neto A , Soares FMC , Pitta F , Furlanetto KC . Functional tests for adults with asthma: validity, reliability, minimal detectable change, and feasibility. J Asthma. 2022;59(1):169‐177. 10.1080/02770903.2020.1838540 33066708

[59] Mori L , Prada V , Signori A , et al. Outcome measures in the clinical evaluation of ambulatory Charcot‐Marie‐Tooth 1A subjects. Eur J Phys Rehabil Med. 2019;55(1):47‐55. 10.23736/S1973-9087.18.05111-0 29898585

[60] Lage VKS , Silva GP , Lacerda ACR , et al. Functional tests associated with sarcopenia in moderate chronic obstructive pulmonary disease. Exp Rev Respir Med. 2021;15(4):569‐576. 10.1080/17476348.2021.1850276 33197358

